# State of Food and Nutritional Security of Urban Households in Grand Lome: Approach by Measuring Household Indicators

**DOI:** 10.3390/foods13213345

**Published:** 2024-10-22

**Authors:** Penagui Toumoudagou N’oueni, Kossiwa Zinsou-Klassou, Jérôme Chenal

**Affiliations:** 1 Regional Center of Excellence on Sustainable Cities in Africa (CERViDA), University of Lome, Lome 01BP1515, Togo; damaguy2000@yahoo.fr; 2 CEAT (Territorial Planning Study Community) and Excellence in Africa, Swiss Federal Institute of Technology in Lausanne, 1005 Lausanne, Switzerland; jerome.chenal@epfl.ch; 3 Center of Urban Systems (CUS), Mohammed VI Polytechnic University (UM6P), Ben Guerir 43150, Morocco

**Keywords:** Grand Lome, demographic and spatial dynamics, purchasing power, food and nutritional insecurity, households

## Abstract

In West Africa, the number of people facing food crises increases each year in both rural and urban areas, due to interdependent factors. The city of Grand Lome in Togo faces an increasingly large population that must ensure access to food, which it hardly produces anymore, because of the establishment of housing and infrastructure. In addition, the increase in the price of food products in this city further weakens the purchasing power of city dwellers in the context of poverty. The objective of this article is to determine the state of food and nutritional security of households in Grand Lome. The methodology adopted is based on documentary research and field surveys. A total of 310 households were interviewed on questions allowing the construction of indicators of food and nutritional security. The results show that despite the availability of food in urban markets, the food situation of households in Grand Lome is not very good. Nearly 49% of households do not have good dietary diversity, and 48% have adopted stress and crisis strategies to access food. Similarly, in Grand Lome, the prevalence of global acute malnutrition is 2.6% of children under 59 months.

## 1. Introduction

One of the United Nations’ Sustainable Development Goals (SDG2) is to end hunger, achieve food security, and improve nutrition by 2030 [1,2]. But the State of Food Security and Nutrition report shows that food insecurity is affecting more and more people worldwide in 2023, from 7.9 percent in 2019 to 9.2 percent of the world’s population in 2022 [3]. In the West African region, the situation of food insecurity and malnutrition continues to deteriorate from year to year. Indeed, the number of people experiencing food insecurity has more than doubled, from around 22.1 million in 2020 to 35.3 million in March 2024 [4]. The same sources indicate that if appropriate measures are not taken, 49.5 million people could be affected by food and nutrition insecurity by the lean season in June–August 2024 [5,6]. This situation results from the complex interactions of various factors such as climate change, increases in food prices, the economic situation, conflicts in certain countries in the region leading to the displacement of populations and loss of their livelihoods, and demographic and spatial dynamics. The results of the work by [7] confirm that the deterioration of the food and nutrition situation in West Africa is mainly linked to the combined effects of conflicts that have exacerbated the adverse effects of climatic shocks, recurrent droughts, inflation, and many other factors that threaten to reduce agricultural productivity and structural factors. The last urbanization factor concerns the population growth coupled with the spatial growth occurring at the expense of agricultural land. In addition, the urban dynamics occur in a general context of poverty, unemployment, and underemployment, which are worsening in all the cities of sub-Saharan Africa [8,9,10], leading to a deterioration in the overall conditions of access of households to basic foodstuffs, especially the most vulnerable households. However, ensuring food security also constitutes an essential basis for promoting sustainable urbanization [11,12,13].

Like most countries in the West African region, Togo’s food security situation is not satisfactory enough. According to the results of the harmonized framework analysis, the country has often experienced food crises since 2020. A total of 35,000 people were in emergency situations in 2022 and this number rose to 40,000 people in 2023, due to the low income of the population, inflation in food prices, and the effects of civil insecurity in the Savannah Region [6]. This situation deteriorated during the lean season following the decrease in food stocks and the seasonal increase in the prices of basic food products. With a population of 8,095,498 inhabitants in 2022, Togo is facing unprecedented population growth over the last 10 years [14]. According to the same source, from 37.7% in 2011, the country’s urbanization rate rose to 43.5% in 2019. This demographic growth is often considered a factor of economic growth. In terms of food in particular, cities are known to experience less seasonality of supply and have better availability and greater food diversity, compared to rural areas. As a result, stakeholders involved in the issue of food security are too rarely interested in the urban environment. However, in cities, food insecurity is masked by aggregated statistics that do not take into account the significant disparities in social, economic, and food situations, characteristic of the urban environment [15,16].

In Grand Lome, the spatial spread of urban fabrics is increasing land pressure on urban and sub-urban agricultural areas. Land that was once used for agriculture is now being taken over by housing and infrastructure.

Indeed, thanks to natural increase and migration, its population has grown at a dizzying pace. In 1897, the Lome conurbation had a population of 2000, rising to 390,000 in 1981 and 2,188,376 in 2022 [14]. The same sources reveal that the Lomé conurbation accounts for 63% of Togo’s urban population, with an average annual growth rate of over 2.84%. This population explosion and the civilization of the “home” that particularly motivates Lomeans and Togolese in general, in a context of horizontal spatial growth, have boosted Lomé’s surface area, which has overflowed its administrative limits set by decree n°71/63 of 1 April 1971 [17]. This demographic and spatial growth has left behind a number of problems that the population must try to resolve or overcome on a daily basis. For example, because of land pressure, only a small handful of the inhabitants of the Lome conurbation have plots of land, bought or rented in outlying areas, on which they farm. As a result, domestic agricultural production of food products in Grand Lome has declined drastically, to the point of being almost non-existent. This is borne out by reports from agricultural campaigns, which no longer mention food production in the city [17]. 

Production and supply zones are therefore increasingly remote, with food supply and distribution systems that are often poorly organized and inefficient. Under these conditions, even if food products are apparently available on the markets, the costs of the latter rise and have a negative impact on the purchasing power of the urban consumer, who is weakened by difficult structural economic conditions. As a result, the problem of food insecurity has much more to do with the accessibility of foodstuffs than with their availability and stability. The economic inability of households, especially poor ones, to access sufficient healthy food is the result of a decline in purchasing power, exacerbated by inflation and economic activity weakened by the lingering effects of COVID-19. In line with this [18], a study on food security has shown that the consequences of the pandemic have particularly aggravated the situation and wiped out the progress painstakingly made in reducing the food and nutrition crisis, due to the disruption of supply systems, particularly in towns. 

As a result, each social class is developing coping strategies to preserve their level of food consumption, especially by reducing the quantity of animal proteins. In this respect, ref. [19] has shown that the food strategies adopted by households are a function of several factors, namely residence in informal settlements, large household size, poverty, low level of education of the head of household, and his or her advanced age. Within households, some children are also affected by malnutrition, resulting in reduced physical, mental, and intellectual capacities [6]. The existing literature does not provide an overview of the food and nutritional situation of households in Grand Lome. It is therefore imperative to assess the state of food and nutrition security in this urban context, in order to have a reference for sustainable development of urbanization and improvement in household food and nutrition security in Grand Lome and in Togo in general.

According to the 1996 World Food Summit, food security is understood as “the physical and economic access for all human beings, at all times, to sufficient, safe and nutritious food, enabling them to meet their dietary needs and food preferences for an active and healthy life”. The concept integrates the four components of food and nutrition security such as the concepts of availability, physical and economic access, which are inseparable from poverty, and food utilization, which is closely linked to nutrition and stability [20,21,22]. Furthermore, while an adequate food supply is essential, the quality of the food available affects the overall well-being of individuals. Therefore, the concept of food security and nutritional security must be addressed simultaneously, taking into account the impact of one on the other, in order to have a healthy population [23,24,25,26].

Given its multidimensional nature, it is not easy to find a single indicator that can establish the state of food and nutritional security of households [27]. Several authors have analyzed food and nutrition security based on factors related to the components of food and nutrition security. These include food availability at local, national, and regional levels, mechanisms for distributing food/agricultural products in time and space, distribution channels, sales prices, income, community support, and food habits and choices [28,29,30,31]. Similarly, according to [21], in 2000, the Committee on World Food Security defined seven (07) indicators for the analysis and monitoring of food security, which are mostly designed to assess food insecurity at the community, national, or international level, but they do not allow the identification of food situation at the household level. Although some studies have focused on indicators related to the experience of food insecurity and the strategies implemented by households, household nutritional indicators have not been taken into account [32,33].

Therefore, for this study, food insecurity was assessed using indicators of food and nutrition security at the household level, mainly the food consumption score (FCS), the share of household food expenditure, the Household Dietary Diversity Score (HDDS), the Food Coping Strategies Index (FCSI), and the Livelihood-Based Coping Strategies Index (LBCSI) as food security indicators. Regarding the measurement of nutritional status, anthropometric indices measuring global acute malnutrition by MUAC were used as indicators [34,35]. The study from a scientific point of view is a contribution of knowledge on the improvement in food and nutritional security of urban households in terms of highlighting the main determining factors that can negatively impact food and nutritional situation and the use of indicators of food and nutritional security at the household level to characterize the state of food and nutritional security of urban households because until now, no study has addressed the issue systematically in Grand Lome.

In view of all these considerations, the general objective of our paper is to determine the state of food and nutritional security of households in Grand Lome. From this general objective, the following specific objectives arise:

▪ Identify the factors influencing food and nutritional security in Grand Lome;
▪ Assess household food security indicators in Grand Lome;
▪ Analyzing indicators of the nutritional status of children under 5 years old in Grand Lome.

## 2. Materials and Methods

### 2.1. Materials

The study material concerns both the study area and the tools for collecting, processing, and analyzing data.

#### 2.1.1. Study Area

**Figure 1:**
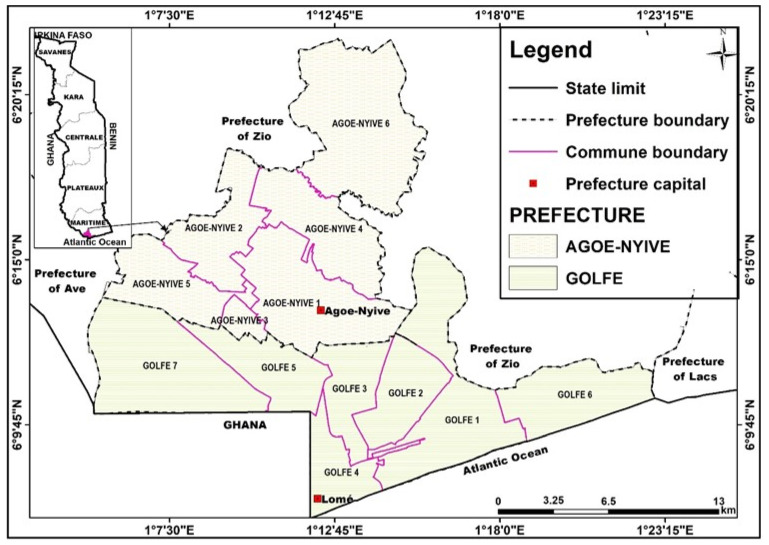
Presentation of the study area. Source: Produced from data from the General Directorate of Urban Planning, 2022.

Located in the extreme southwest of the country, precisely in the District of the Golfe and that of Agoè-Nyivé, which themselves are located in the Maritime region, Grand Lome is located on the coast of the Atlantic Ocean between 6°6′27 and 6°20′32 north latitude and between 1°4′50 and 1°22′25 east longitude. The agglomeration of Grand Lome extends along the coast of the Gulf of Guinea over a distance of more than 20 km, and today covers an area of more than 335 km² (Figure 1). This study area includes two districts (Golfe and Agoè-Nyivé) and thirteen municipalities, namely Golfe 1, Golfe 2, Golfe 3, Golfe 4, Golfe 5, Golfe 6, Golfe 7, Agoè-Nyivé 1, Agoè-Nyivé 2, Agoè-Nyivé 3, Agoè-Nyivé 4, Agoè-Nyivé 5, and Agoè-Nyivé 6. According to the results of the 5th General Population and Housing Census [14], Grand Lome is the most populated agglomeration in the country with a total population of 2,188,376 inhabitants, including 1,060,504 males and 1,127,872 females.

The main economic activities are trade and industry. Given its urban character, Grand Lome for its food supply depends almost entirely on the other regions of the country or imports from other countries. Cereals are the staple food of the population whose base prices are still up 39% in March 2024 compared to last year and the five-year average [36].

The choice of this area can be justified by its full mutation from a demographic point of view and from the point of view of the spatial extension of the urban perimeter through the saturation of housing, infrastructure, and especially in the increase in the cost of supplying food products. In addition, the food and nutritional security analyses that are carried out each year at the level of the Ministry of Agriculture of Togo in collaboration with the actors of the sector do not take into account Grand Lome, considering that it is the capital of the country and therefore, there will not be a major problem of food and nutritional insecurity.

#### 2.1.2. Data Collection, Processing, and Analysis Tools

The tools are essentially those for collecting, processing, and analyzing data.

For primary data collection at the household level, an individual questionnaire was used through the KoboCollect application. The questionnaire included the following sections relating to households: (i) location; (ii) socio-demographic characteristics of respondents; (iii) household income; (iv) food consumption score; (v) food diversity score; (vi) household food expenditure; (vii) coping strategies based on household diet; (viii) coping strategies based on household livelihoods; and (viii) anthropometric data for children aged 6–59 months.

To collect anthropometric data from children aged 6 to 59 months, in addition to the questions asked, a tape measure (MUAC) was used to measure the brachial perimeter.

In addition, a semi-structured interview guide was used to frame discussions/exchanges with key informants. 

Statistical analysis was performed using SPSS software. Microsoft Office Excel was used to produce the statistical graphs, and QGIS software was used to produce the presentation map of the study area. 

### 2.2. Determining Sample Size and Drawing the Sample

Data collection was carried out using a qualitative approach (documentary research, interviews with resource persons, consultations on certain websites) and a quantitative approach (household surveys).

#### 2.2.1. Calculate the Optimal Sample Size

To determine the sample size, the Schwartz formula was used to estimate the minimum size needed to ensure, under certain conditions (including random selection), the appropriate representativeness of the data/results at the stratum level. The Schwartz formula is as follows:

$$n=\frac{z^{2}*p*\left(1-p\right)*k*(1+TNR)}{d^{2}}$$

Considering the Schwartz formula,

- A 95% confidence level (in this case, z = 1.96);
- The recent prevalence of households with a poor and borderline food consumption score (DSID-CILSS survey, September 2022) is 25% for Agoé-Nyivé and 30% for the prefecture of Golfe;
- A cluster effect (k) of 1.5; a value similar to those of other surveys of this type carried out in Togo and which is quite close to the default value (2) most often recommended in classic surveys;
- A desired minimum accuracy (d) of 8.5%;
- A TNR of around 2% similar to that of other surveys of this type carried out recently in Togo.

By replacing the parameters with their value in the formula, the total sample size is 310 households, including 152 households for the prefecture of Agoé-Nyivé and 158 for that of Golfe.

Although the Schwartz formula, a reference for size determination, was used to estimate the minimum size necessary to ensure, under certain conditions (including random selection), appropriate representativeness of the data/results at the level of each prefecture and Grand Lome, the following limitations must be taken into account:

- Necessary readjustments have been made to certain parameters (minimum precision instead of 5%, non-response rate 2% instead of 0%), taking into account available resources;
- Due to the non-proportional distribution of the sample among the different strata or areas of interest for this survey and taking into account the different response rates to the survey by stratum, sampling weights are necessary in all analyses to ensure that the sample is representative of Grand Lome.

#### 2.2.2. Sample Drawing

For the selection of survey units, a three-stage sampling was carried out. The first stage corresponds to the selection of the municipalities of the Grand Lome agglomeration. Overall, 50% of the municipalities were selected, i.e., 7 municipalities were selected out of a total of 13. The selection criterion was based on the geographical location of these communes, i.e., whether they were central or peripheral to each of the prefectures. This was performed to ensure a more representative sample or a better meshing of the study area.

The second stage concerns the selection of districts, which was conducted by randomly drawing 30% of the districts of each municipality. In total, the survey was conducted in 42 districts including 20 districts in the prefecture of Golfe and 22 districts in the prefecture of Agoè-Niyvé (Table 1).

Similarly, at the third level, households were selected in a simple random manner. A prior list of all urban households in the study area divided by municipality and respective district was not available for the identification of respondents, and the use of “sample steps” during the collection of data in the field was made [37]. In the field, at the neighborhood level, the first household was chosen arbitrarily and randomly and surveyed. The next household was chosen by applying a step of 10en from the first and so on until the quorum expected for the zone was reached [37].

Table 1, below, shows the distribution of the sample by prefecture, by municipality, and by district.

Field investigations revealed 303 children aged 6 to 59 months in Grand Lome; 137 of them were male, compared with 166 females in some of the households surveyed. At the prefectural level, 151 children aged 6 to 59 months were recorded in the Agoe-Nyive prefecture, compared with 152 in the Golfe prefecture. Thus, the representative number of children (150) per prefecture was reached.

### 2.3. Data Collection, Processing, and Analysis

This section highlights the way in which quantitative and qualitative data were collected. The quantitative data collection operation was carried out by ten (10) interviewers and five (5) supervisors. Data collection was carried out using tablets, with the questionnaire programmed for this purpose using the KoboCollect application. The structured household questionnaire was administered a priori to the head of household or, failing that, to the respondent (member of the household) most knowledgeable and therefore able to provide the information sought. 

In addition, interviews were conducted with twelve (12) key informants, including officials from the various town halls, technical structures of the Ministry of Agriculture, experts from regional structures involved in food and nutritional security, restaurant managers, food transporters, and agricultural product processors. These interviews enabled us to gather information on the context of household vulnerability to food and nutritional insecurity in the study area. 

The data stored on the dedicated Kobo server were exported in Excel format and then to SPSS for processing in line with the processing plan drawn up for this purpose. The data from these interviews were transcribed, entered, and compiled using a tabulation grid.

SPSS software was used to generate all indicators, cross-tabulate variables, and perform other statistical tests. QGIS software was used to map the study area.

To ensure the validity and reliability of the data, a data quality control and assurance mechanism was set up, based on

- An effective system of close supervision of field agents’ work;
- Rigorous monitoring/control of the quality of the raw data collected and transmitted daily to the Kobo server. By centralizing the data collected on the Kobo server, it was possible to carry out analyses in near-real time and to carry out daily checks on the consistency and quality of the questionnaires/interview guides filled in—all the while assessing the performance of the agents in the field, as well as the overall progress of the data collection work in line with the provisional schedule of activities.

### 2.4. Construction of Indicators

The indicators constructed to characterize the food and nutrition security of households in the study area include the Food Consumption Score (FCS), the Household Dietary Diversity Score (HDDS), the Share of Food Expenditure, the Food Coping Strategies Index (FCSI), the Livelihood-Based Coping Strategies Index (LBASI), and anthropometric indicators such as global acute malnutrition (GAM/MUAC), moderate acute malnutrition (MAM/MUAC), and severe acute malnutrition (SAM/MUAC).

#### 2.4.1. Food Consumption Score (FCS)

The Food Consumption Score is a composite score of the past 7-day diet based on dietary diversity (number of food groups consumed by a household during a 7-day reference period), frequency of consumption (number of days each food group is consumed), and the relative nutritional importance of different food groups.

The calculation of the food consumption score is as follows:

$$SCA=\left(a_{aliments\;de\;base}*X_{aliments\;de\;base}\right)+\left(a_{légumineuses}*X_{légumineuses}\right)+\left(a_{légumes}*X_{légumes}\right)+\left(a_{fruits}*X_{fruits}\right)\;+\left(a_{huile}*X_{huile}\right)+\left(a_{viande\;et\;poisson}*X_{viande\;et\;poisson}\right)+\left(a_{lait}*X_{lait}\right)+\left(a_{sucre}*X_{sucre}\right)$$

SCAFood Consumption ScorexiFood consumption frequencies = number of days that the food was consumed in the last 7 days (for the same food group, this number cannot exceed 7)haveWeight of each food groupSource: CILSS, 2019.

Once the SCA is calculated, thresholds for groups should be determined based on the frequency of scores (Table 2).

#### 2.4.2. Household Dietary Diversity Score (HDDS)

The diversity of food consumption measures the number of different foods or food groups consumed during the 24-h recall period. It is constructed on the basis of a list of food groups (SDA). For each group considered, a point is awarded if the group had been consumed the day before by the subject interviewed. The diversity score corresponds to the sum of the points awarded to the 12 groups considered. The SDA that was calculated corresponds to the dietary diversity score proposed by the FAO in 2013.

Calculation of the household dietary diversity score (HDDS)

- Group all food products into specific groups if necessary;
- For each group, create a new binomial variable that is equal to 1 if the household/individual consumed the given product group and 0 if not;
- Add up the values of the different groups to obtain the HDDS;
- The new variable should range from 0 to the maximum number of 12 product groups collected.

$$SDAM=\sum P_{i}$$

*SDAM* = Dietary diversity score

*P_i_* = 1 if the food group is consumed and 0 if not

The interpretation of the SDAM is presented in Table 3:

#### 2.4.3. Share of Food Expenditure

Economic vulnerability is measured using the indicator “share of food expenditure”. This indicator is based on the assumption that the greater the share of the budget devoted to food within a household budget (relative to other goods/services consumed), the more economically vulnerable the household is.

Monthly household food expenditures are calculated by taking into account the previous day’s food expenditures (meat, fish, milk, meals, other foods) brought forward to the month, the weekly food expenditures (fish, milk, other foods) also brought forward to the month, and the monthly food expenditures (processed and stored cereals, other foods purchased each month). The total monthly household expenditures are calculated by taking into account food expenditures, clothing, rent, water, electricity, gas, furniture, health, transport, leisure, soap, other goods and services, and education. Variables recorded on an annual basis are brought forward to the month. Food expenditures and total expenditures per person are calculated by dividing food expenditures or total expenditures by the number of people in the household. The share of food expenditures is constructed by dividing the monthly household food expenditures by the total monthly household expenditures, all multiplied by 100.

However, the measurement of economic vulnerability mainly focuses on how much (as a proportion) of total household expenditure is spent on non-food items. In other words, to what extent food is more important than non-food items. For this purpose, the indicator is appropriate for classifying households with different food acquisition patterns [38].

Ranking of the indicator “share of expenditure and its interpretation”

Table 4 shows the ranking of the indicator “share of food expenditure” and its interpretation.

#### 2.4.4. Dietary Coping Strategies Index (rCSI)

The Food Coping Strategy Index (rCSI) is a simple indicator to use to assess the food security situation of a household. It consists of the different strategies that households use to cope with food difficulties. They are asked to specify these strategies and the number of days they would have used them during the seven (7) days preceding the collection.

The higher the rCSI, the more the household relies on more severe coping strategies to cope with its difficulty in accessing food. Each strategy has a corresponding weight and the Weighted Score is obtained by multiplying the number of days by weight.

The interpretation of the rCSI is as follows (Table 5):

#### 2.4.5. Livelihood-Based Adaptation Strategies Index (LBASI)

The Livelihood Strategies Index indicator is derived from a series of questions regarding household experience of livelihood strategies that may lead to asset depletion in the 30 days preceding the survey. The responses are used to understand the different levels of adaptation describing households’ capacities to manage shocks based on their assets.

➢ Indicator estimation approach—ISAME

First, a set of 10 coping strategies from the previous lists is chosen. This choice of strategies is made as follows: 4 stress strategies; 3 crisis strategies; and 3 emergency strategies.

Then, each household is classified according to the most severe strategy category it used (Table 6).

#### 2.4.6. Anthropometric Indices

Anthropometric indices measuring global acute malnutrition by MUAC from representative surveys were calculated for children aged 0 to 59 months. The calculation principle was conducted automatically with specific software (ENA).

➢ Classification of the severity of acute malnutrition

- Moderate acute malnutrition (MAM): <−2 z-score and >=−3 z-score, without edema;
- Severe acute malnutrition (SAM): <−3 z-score and/or edema;
- Global acute malnutrition (GAM): <−2 z-score and/or edema is the sum of the two degrees of acute malnutrition in the population: MAM and MAS = MAG.

Table 7 shows the classification of acute malnutrition by mid-upper arm circumference (MAG/MUAC).

## 3. Results

### 3.1. Factors Influencing Food and Nutrition Security

#### 3.1.1. Main Shocks Suffered by Households

During the last three (3) months preceding the field work, households suffered significant shocks.

The survey results indicate that households were much more affected by shocks related to illness or death of an active member of the household (65.5%), rising food prices (57.7%), loss of employment or inability to earn a living (40%), rising fuel prices (20%), flooding (7.7%), fire (3.2%), and theft (1%). 

Indeed, when the head of the household or an active member falls ill, it reduces the household’s ability to mobilize the financial resources or livelihoods needed to acquire food. In addition, rising food prices force households to spend more, which negatively impacts their purchasing power and limits their ability to cope with difficulties in accessing food. This situation also applies to other hazards that households report having faced. 

The intensification of these social, economic, and natural factors is interdependent, aggravating the fragility of food systems, disrupting the socio-economic fabric, and amplifying the vulnerability to food and nutritional insecurity of a growing fringe of the urban population.

**Figure 2:**
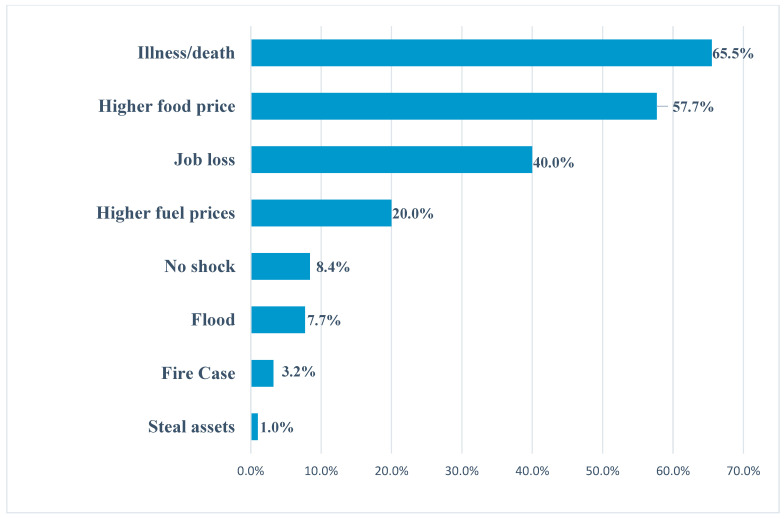
Major shocks suffered by households over the last three months. Source: Field survey, November 2023.

Figure 2 shows the main shocks experienced by households in the three months preceding the survey.

#### 3.1.2. Income Drop for the Majority of Households in Grand Lome

Over the past five years, household incomes have generally declined. In fact, more than half (54%) of the households surveyed experienced a drop in their income, while nearly a third (27%) reported having observed no change, while 19% noted an increase in their income. This deterioration in the economic situation has affected the purchasing power of households in Grand Lome, particularly those whose main activity is commerce (70.0%), followed by liberal professions such as lawyers, surveyors, bailiffs (66.7%), transport professionals (in particular motorcycle taxi drivers (64.5%), retirees (57.1%), and professions such as tailors, dressmakers, and hairdressers (54.6%). It is important to note that the drop in income is particularly marked among households whose main activity is in the informal sector.

In addition, a higher percentage of households experiencing a drop in income was observed in Golfe prefecture (55%) compared to Agoè-Nyivé (50%). Taking into account the gender of the household head, approximately 68% of households headed by women experienced a drop in income, compared to 49% for those headed by men.

Low or declining incomes are one of the factors contributing to high levels of food and nutritional insecurity in urban areas. Indeed, the economic inability of especially poor households to access food has resulted in a decline in purchasing power, exacerbated by food price inflation and weakened economic activity. These economic challenges were compounded by the country’s structural poverty, low economic growth, and the lingering effects of COVID-19.

This decrease in income has led to a reduction in purchasing power, thus negatively affecting household food and nutrition security. Faced with these challenges, households generally rely on the assets they own to cope with the shocks related to the food insecurity they experience. However, the depletion of these assets makes them more vulnerable to future shocks. The analysis of livelihood-based coping strategies is essential to better understand the extent of this asset depletion.

Table 8 shows the evolution of the income of the households surveyed according to the main activity, the place of residence, and according to the sex of the head of household.

### 3.2. Measurement of Household Food Security Indicators

Food security analysis is carried out using several indicators, including the main food groups consumed by households, the food consumption score, the food diversity score, the share of their income spent on food, and the coping strategies used by households to acquire food (the reduced food strategy index and coping strategies based on household livelihoods).

#### 3.2.1. Main Food Groups Consumed by Households and Their Origin

This section covers the main food products consumed by households during the seven days preceding the survey and provides them with the energy and nutrients their bodies need to lead healthy and active lives.

Field work shows that in Grand Lome, cereals and tubers constitute the basis of household diets (98.1%), followed by protein-rich products (92.3%), oils (90.3%), vegetables (87.4%), and sweet products (61.9%). On the other hand, it is noted that dairy products (25.5%), fruits (38.8%), and legumes (42.6%) are consumed by less than half of households. These results highlight the eating habits of households that are oriented toward cereals and tubers, proteins, vegetables, and oils unlike dairy products, fruits, and legumes.

As for the source of supply of these foods, the main method of acquiring these food products remains purchase (96.64%) in the markets or shops of Grand Lome.

**Figure 3:**
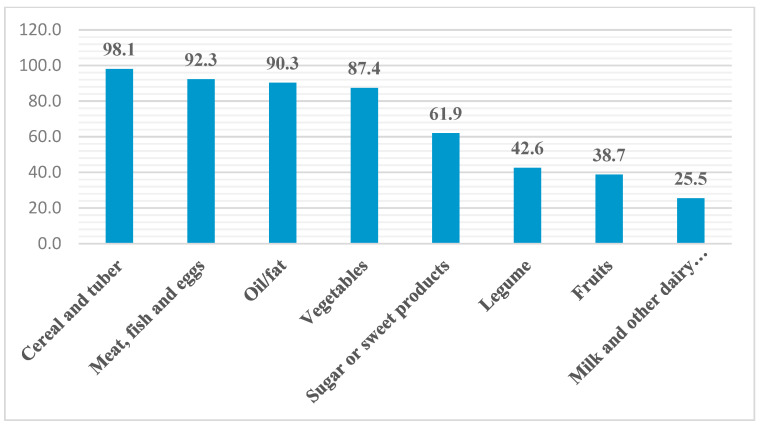
Proportion of households according to consumption of different food groups. Source: Field survey, November 2023.

Aside from eating habits, it is important to note that with the low purchasing power of certain segments of the population, the non-consumption of dairy products, fruit, and legumes could be linked to the fact that most people in Grand Lome want to fill their bellies first with staple foods made from corn, rice, and yams, rather than buying milk, fruit, and cowpeas, which are often considered non-essential and expensive. Yet, these food groups, which are not regularly consumed by the population, contain the nutrients needed for an active healthy life. Figure 3 below shows the proportion of households according to their consumption of different food groups.

#### 3.2.2. Acceptable Food Consumption Score in Grand Lome

The food consumption score was calculated using data on the types of food usually consumed, grouped into standard food groups, and the frequency of use of these foods. It provides information on the current food situation of households over the last 7 days prior to the survey.

Field surveys show that the majority of households in Greater Lomé have an acceptable consumption score (73.9%). However, 26.1% of urban households do not have a good food consumption score, including 3.2% with a poor consumption score.

In fact, the results show that the Golfe prefecture (36.1%) is home to more households with poor food consumption than the 15.8% of households in the Agoè-Nyivé prefecture. This could be explained by the fact that more households in the Golfe prefecture experienced a drop in income than those in the Agoè-Nyivé prefecture. Furthermore, analysis of the link between food consumption score and household socio-demographic characteristics shows that prefecture of residence and exposure to shocks have a significant impact on household food consumption.

Furthermore, the results show that 25.8% of female-headed households do not have a good food consumption score, compared with 26.2% of male-headed households. However, this difference does not appear to be significant. In addition, the gender of the head (decision-making center) of the survey unit may be a factor predisposing certain households to food insecurity. It is often assumed that female-headed households are more vulnerable to food insecurity than male-headed households. 

Although the age variable is not significant, we find that the risk of low consumption decreases with age and stagnates after the age of 54. Similarly, households that have been married once (married, divorced, and widowed) appear to have better food consumption than single households. Indeed, analysis of these results suggests that as the head of household advances in age, he or she has time to better build up his or her means of existence (training, skills, wages, access to credit, savings, motorcycle, car, house, membership of religious groups/associations, etc.). This will enable them to generate more income, which in turn will help protect their households from food insecurity.

The principal activity and the existence of a second activity of the head of household do not influence food consumption in urban households. However, formal employment (civil servants 91.7%, private sector agents 78.1%) seems to guarantee better household food consumption. The activity carried out by the head of the household offers a perspective on the various sources of household income and their implications for the household’s ability to access food. Households with precarious activity are particularly vulnerable to fluctuations in income and food prices (see Appendix A Table A1).

Table 9 shows the proportion of households with their food consumption score.

#### 3.2.3. Low Dietary Diversity of Households in Grand Lome

The Dietary Diversity Score (DDS) assesses the different food groups consumed during a 24-h reference period preceding the survey. It is an indirect indicator used as a proxy measure of household socioeconomic status.

Overall, the results show that more than 2/3 of households (72.1%) do not have good dietary diversity, of which 60.3% of households have average dietary diversity (four to five food groups) and 11.9% of households have poor dietary diversity (one to three food groups). On the other hand, 27.7% of households have good dietary diversity (more than five food groups). This situation is mainly explained by eating habits oriented toward cereals and tubers accompanied by sauces (vegetables, oils, and meat/fish) with low use of dairy products, legumes, and fruits. 

Of all the characteristics analyzed, only education is a determining factor in dietary diversity. Household heads with Islamic education (50%) and those with university education (43.1%) appear to have better dietary diversity. This result implies that a household head’s low level of education is a sign of vulnerability. Indeed, an uneducated head of household is exposed to marginalization and cannot actively participate in the decision-making processes of the society in which he or she lives and play a role as a free citizen. Moreover, this could have adverse effects on the nutritional status of children under 5, especially in female-headed households.

The surveys also show that households headed by women (77.4%) and those headed by men (71%) do not diversify their diet in Greater Lomé. Households do not have a good food consumption score, compared with 71% of male-headed households.

The prefecture with the most households with low dietary diversity is Agoè-Nyivé (72.4%), versus 72.2% of households in the Golfe prefecture. However, it is important to note that this difference is not significant between prefectures. 

Despite the fact that the difference is not significant, households headed by married (28.9%) or divorced (26.9%) persons have a greater propensity to diversify their diet than households headed by single (23.8%) or widowed (22.6%) persons (see Appendix A Table A2). 

It is important to note that a poorly diversified diet does not cover all the body’s needs, leading to deficiencies in specific nutrients, which could weaken the immune system. 

Table 10, below, shows the proportion of households according to their dietary diversity score.

#### 3.2.4. Share of Food Expenditure

The share of food expenditure in total household expenditure is used as an indicator of economic vulnerability. The assumption is that the higher the share of total expenditure on food, the less able households are to meet non-food needs and have less capacity to cope with shocks. When the share of food expenditure is

▪ Below < 50%, the households concerned are food secure;
▪ Equal to 50% and less than 75%, the households concerned are classified as moderately food insecure;
▪ Greater than or equal to 75%, the households concerned are classified as severely food insecure.

Analysis of the results from field investigations shows that overall, approximately 43% of households spend less than 50% of their budget on food expenses. This result indicates that more than two in five households are in a situation of food security. However, 44% of households spend between 50% and just under 75% of their income on food expenses, which means that these households are in a moderate insecurity situation. In addition, 13% of households spend more than 75% of their budget on food expenses, which means that these households are in a situation of severe food insecurity.

These results show that more than half of households (58%) spend 50% to more than 75% of their budget on food expenses. This is explained by the fact that urban populations depend almost exclusively on markets for their food and non-food supplies. In addition, the situation may have been aggravated by the exceptional increase in food prices on the markets. This situation makes them very sensitive to changes in food prices, as evidenced by the difficulties experienced by households in Grand Lome following the COVID-19 health crisis and the Russo–Ukrainian war.

Thus, within these households, the greater the proportion of expenditure on food, the greater the risk of food insecurity. A household whose financial resources are mainly devoted to food has a high risk of food insecurity. It also has a very low capacity to resist the onset of a shock, especially if it affects livelihoods.

As a result, it seems to be more difficult for households devoting 50% to over 75% of their budget to food expenditure to achieve a much more balanced diet, as nutritious foods (fruits, legumes, and fruits) are relatively expensive compared to staple foods such as cereals and roots and tubers (energy-rich foods). 

**Figure 4:**
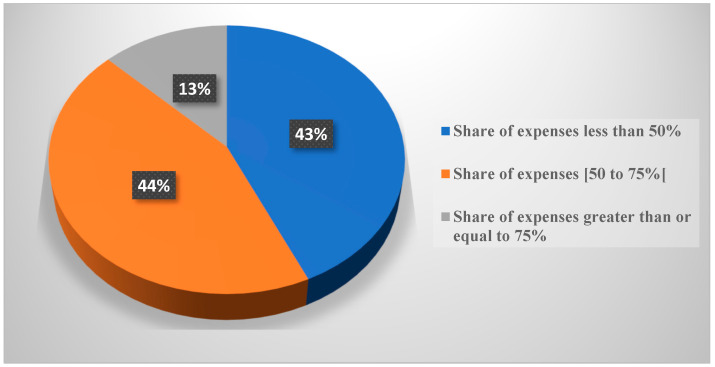
Share of food expenditure in total expenditure. Source: Field survey, November 2023.

Figure 4 shows the proportion of households according to their share of food expenditure in total expenditure.

#### 3.2.5. Implementation of Adaptation Strategies by Households in the Face of Food Insecurity

Household coping strategies are a key component of food security outcomes analysis. They show the ways and means by which households manage and cope with food difficulties. These strategies provide information on the severity and extent of household behaviors when faced with food and/or financial deficits.

##### Reduced Food Strategy Index (rCSI) of Households in Grand Lome

This section deals with the different food-based strategies that households had to resort to in order to cope with food difficulties. They were asked to name these strategies and their frequency of use during the last 7 days preceding the survey.

According to the results of the field surveys, households mainly resort to food strategies such as buying the least preferred and least expensive foods (58.1%), limiting the portion eaten at each meal (35.2%), reducing adult consumption to allow young children to eat (34.2%), and reducing the number of meals eaten in a day (30%). Thus, in a context of rising food prices (inflation), we understand why households in Grand Lome indulge in the consumption of less nutritious foods (cereals and tubers), which are at the origin of the rise in chronic diseases linked to poor nutrition (undernutrition, obesity, type 2 diabetes, etc.).

**Figure 5:**
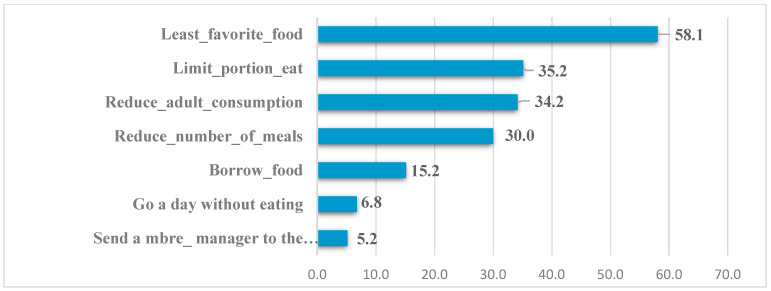
Proportion of households according to the food strategies adopted. Source: Field survey, November 2023.

The following Figure 5 shows the proportion of households according to the food strategies adopted.

The above-mentioned different food strategies that households used during the last seven days preceding the survey were used to calculate the reduced Food Strategies Index (rCSI). The higher this index (rCSI), the more the household relies on more severe survival strategies to cope with its difficulty in accessing food.

In view of the results (Table 11), almost half of the households (48%) in Grand Lome have difficulty accessing food. Indeed, 39% of the households surveyed are in a food stress situation and 9% adopt crisis strategies. On the other hand, 51.9% of households use strategies considered minimal to obtain food.

This high proportion of households relying on stress and crisis survival strategies to cope with their difficulty in accessing food could be linked to the inflation of food prices in urban markets, which leads households to devote more of their income to purchasing food.

Analysis of the relationships between food-based strategies and socio-demographic characteristics shows that area of residence (prefecture), household size, level of education, and main activity of the head of household are significant at the 1% threshold and at the level of education at 5%.

The prefecture with the highest proportion of households adopting crisis and stress strategies is Golfe (53.8%), compared with 42.1% in Agoè-Nyivé. This difference is significant at the 10% level.

Indeed, the larger the household size, the more households develop strategies to access food. The proportion of households with a minimum situation varies from 60% in households of 1 to 3 people to 28% in households of more than 10 people. Being a civil servant (87.5%), self-employed (66.7%), or working in the private sector (59.4%) seems to improve access to food. 

An analysis of the level of education shows that the more educated the head of the household, the fewer strategies the household develops to access food. This is due to the fact that uneducated households are mainly blue-collar workers with temporary and precarious jobs, whereas highly educated households have access to formal and more secure jobs. In households where the head of the household has taken Islamic courses, the latter (CM) would be religious leaders (Imam, Marabout) benefiting from donations and other social assistance and therefore do not develop strategies to access food, (see Appendix A Table A3).

When analyzed by gender, female-headed households (54.8%) were found to use more crisis and stress strategies than male-headed households (46.4%). However, this difference in the use of food strategies was not significant. The same applies to the marital status of household heads.

Table 11 below shows the proportion of households according to their reduced Food Strategies Index.

##### Implementation of Livelihood-Based Adaptation Strategies (ISAME) by Households in Grand Lome

Field investigations show that in Grand Lome, during the last 30 days preceding data collection, households referred to their livelihoods to cope with difficulties in accessing food. The livelihood strategies used by households are the following: spending household savings (49.7%), buying food on credit (37.4%), borrowing money (34.2%), selling household non-productive assets (radio, furniture, refrigerator, television, jewelry, etc.), reducing essential non-food expenses such as education and health (19.4%), selling household non-productive assets (8.4%), selling household assets (3.9%), withdrawing children from school (2.6%), migrating an entire family (2%), and engaging in illegal activities such as begging (1%). Spending household savings, buying food on credit, borrowing money, and selling household non-productive assets (radio, furniture, refrigerator, television, jewelry etc.) are signs of stress or mild food insecurity. Whereas, strategies such as reducing essential non-food expenditures, i.e., education and health, selling household non-productive assets, selling household assets, and withdrawing children from school are, therefore, considered crisis strategies or moderate food insecurity. As for strategies such as migrating an entire family and engaging in illegal activities such as begging, these are termed emergency strategies as they are considered to impact future productivity and are more difficult to reverse.

**Figure 6:**
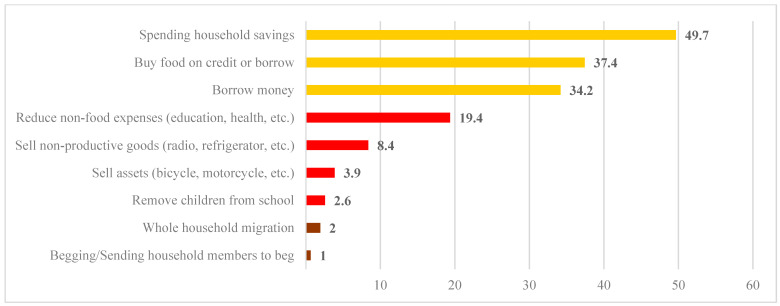
Proportion of households according to survival strategies based on livelihoods (ISAME) in Grand Lome. Source: Field survey, November 2023.

The following Figure 6 shows the proportion of households according to their use of livelihood strategies.

The indicator provides a better understanding of the different levels of adaptation describing households’ ability to manage shocks based on their assets. The more households rely on their assets to cope with the effects of shocks, the more vulnerable they become. The survey results show that during the last thirty (30) days preceding the survey for all respondents (Table 12), 36.1% of households did not use livelihood strategies to feed themselves compared to 63.9% of households that did. Among these households, 40% of households used stress strategies, 21% used crisis strategies, and 2.9% adopted emergency strategies. In short, regarding livelihoods, households develop more stress and crisis strategies to feed themselves. This high proportion of households relying on these stress or crisis coping strategies to cope with their difficulty in accessing food could be linked to the upward trend in food prices on the markets. These strategies adopted by households could threaten the livelihoods of households and their lives in the medium and long term.

A cross-analysis of livelihood strategies with socio-demographic characteristics shows that only the age and main activity of the household head influence the adoption of harmful livelihood strategies at the 5% significance level (see Appendix A Table A4).

### 3.3. Nutritional Status of Children under 5 Years of Age That Is Generally Acceptable in Grand Lome

Nutritional indicators were obtained by measuring the Upper Arm Circumference (MUAC) in children aged 6 to 59 months living in the surveyed households. An Upper Arm Circumference (MUAC) greater than 115 mm and less than 125 mm indicates a state of moderate acute malnutrition (MAM), while an arm circumference less than 115 mm corresponds to severe acute malnutrition (SAM). Children suffering from SAM according to the Upper Arm Circumference (MUAC < 115 mm) run a very high risk of death in the absence of good management. Notably, global acute malnutrition (GAM) includes SAM and MAM.

The results of the field work (Table 13) indicate that in Grand Lome, 2.6% of children under 59 months have global acute malnutrition MUAC <125, including 1.4% who are moderately malnourished (115 mm ≤ MUAC ≤ 124 mm) and 1.2% who are severely malnourished (MUAC < 115 mm). The highest rate of children with global acute malnutrition is in the prefecture of Agoè-Nyivé (2.1%) compared to 1.3% of children in the Prefecture of Golfe. Considering the sex of the child, the field surveys reveal that the prevalence of global acute malnutrition by measurement of the arm circumference (MUAC < 125 mm) in children aged 6–59 months is 2.4% in female children, including 1.8% who are moderately malnourished and 0.6% who are severely malnourished. On the other hand, among male children, the prevalence of global acute malnutrition by measurement of the brachial perimeter is 1.6%, of which 1% are moderately malnourished and 0.6% of children are severely malnourished.

This proportion of children in a state of global acute malnutrition is below the critical threshold, and we can deduce that the food situation at household level in Greater Lomé has an impact on the nutritional situation of children aged 6–59 months, but not on a large scale. Indeed, in young children, poor food quality and dietary diversity increase the risk of suffering not only from nutritional deficiencies, but also from stunted growth, malnutrition, and cognitive and behavioral development disorders. 

A plausibility test was carried out to assess the quality of brachial perimeter measurements collected between the ages of 6 and 59 months. According to the results of this test, the overall score of the survey is 7% in Grand Lome, which is excellent for the reliability of the data according to the WHO 2006 reference for brachial perimeter measurement. 

The following Table 13 shows the prevalence of acute malnutrition in children aged 6 to 59 months by prefecture and in Grand Lome.

## 4. Discussion

### 4.1. Factors Influencing Household Food and Nutritional Security in Grand Lome

The various results obtained reveal that there are factors that influence household food security in Grand Lome. These are mainly shocks suffered by households, which impact the ability to access food and aspects relating to access to drinking water and sanitation.

Indeed, research indicates that households were more affected by shocks related to illness or death of an active member of the household (65.5%), rising food prices (57.7%), loss of employment or inability to earn a living (40%), and rising fuel prices (20%). The illness or death of an active member of the household is a factor influencing household food and nutrition security because first of all, money has to be spent on the person’s care or funeral. Moreover, this member of the household is no longer in a position to provide financial resources for the house. Yet, the majority of the population has no health or death insurance. As a result, households are forced to use up all their resources, or even go into debt, to cope. Rising food prices, meanwhile, are forcing households to spend more than usual on foodstuffs, i.e., on incomes that are stagnating or even falling. This will not only have a negative impact on their ability to acquire the quantity and quality of food they need but also on the foods that meet their dietary preferences. The same applies to job loss or inability to earn, as well as higher fuel prices. This weakens people’s ability to mobilize the necessary financial resources or livelihoods, and leads to a decline in their purchasing power to acquire food. These interdependent and complex factors increase the vulnerability to food and nutritional insecurity of a growing proportion of urban populations, and consequently limit their ability to cope with difficulties in accessing food. Sufficient and diversified food quality that meets dietary preferences is an important basis for the nutritional health of inhabitants, enabling them to lead active healthy lives. This result is similar to that of FAO [7,38] in its study on agricultural livelihoods and food security in the context of COVID-19 in Togo. It showed that rising prices, loss of income, and illness are the shocks most frequently reported by more than two-thirds of respondents. Furthermore, the results showed that more than half of the households surveyed (54%) experienced a decrease in their income. This decrease in income causes a decrease in the purchasing power of households and therefore negatively influences their food and nutritional security. These results corroborate with those of Lian, Y. et al. [39] in their study on the reasonableness and spatial differences in the structure of food consumption of urban and rural residents in China, 2015–2021. 

In contrast, they have shown that the effect of an increase in income is characterized by an increase in the consumption of much more balanced foods (animal products, dairy products, and fruit and vegetables). In response to the effects of these factors influencing the food and nutrition situation of urban households, it would be advisable to reinforce and extend social protection actions to vulnerable households, for example, through health or all-risk insurance to cover the risks linked to the shocks suffered by households, price subsidies for basic food products, and the development of new income opportunities through income-generating activities for unemployed household heads, or to create assets in synergy with social safety net programs for vulnerable households that have experienced a significant drop in their income. In addition, food prices show strong temporal variations (seasonality), underlining the importance of temporal and disaggregated price information at a market level for monitoring food and nutritional security. 

### 4.2. Measuring Household Food Security in Grand Lome

The state of food security is assessed through several indicators. In urban Togolese areas, particularly in Grand Lome, eating habits are more oriented toward the consumption of cereals and tubers (98.1%) accompanied by vegetable sauces (87.4%) composed of meat or fish (92.3%), enriched with vegetable oils (90.3%) and sweet products consumed in the form of drinks and added to porridge (61.9%) to the detriment of dairy products (25.5%), fruits (38.8%), and legumes (42.6%). This propensity to consume food groups such as cereals and tubers, vegetables, meat or fish, vegetable oils, and sweet products is linked, on the one hand, to people’s eating habits. On the other hand, it is important to note that with the low purchasing power of certain segments of the population, the non-consumption of dairy products, fruit, and legumes could be linked to the fact that most people in Greater Lomé want to fill their bellies first with staple foods such as corn, rice, and yams, rather than buying milk, fruit, and cowpeas, which are often considered non-essential and expensive. Yet, these food groups, which are not regularly consumed by the population, contain the nutrients needed for an active healthy life.

This result corroborates those of Allen Thomas and Philipp Heinrigs [40] who have demonstrated that urban households, in their consumption, prefer more fruits and vegetables, meat products, and fish. They estimate that in urban environments, households make substitutions between foods by consuming fewer food groups considered less noble or inferior in favor of calories of value considered higher. On the other hand, the work of Akoua Assunta A. [41] on the assessment of food security in Côte d’Ivoire by the harmonized framework method shows a different situation for rural households. Thus, it demonstrated that cereals, tubers, and vegetables were almost consumed in all households at 89%, during the last seven days, before the survey, unlike meat, eggs, milk, and fruits appearing only episodically in the food consumption of rural households.

This state of affairs results in poor food consumption and low dietary diversity in households. Indeed, the results show that 26.1% of urban households do not have good food consumption, of which 22.9% have borderline food consumption and 3.2% have poor consumption. In addition, about half of households (49%) do not have good dietary diversity (consume one to five food groups). Results from CILSS, FSRP, and DSID [42] obtained as part of the food and nutrition security survey in Togo show a much worse situation. Their field survey shows that 47.9% of urban households do not have good dietary diversity, of which 30.6% have average dietary diversity and 17.3% have dietary diversity. This difference could be explained by the data collection period. Their data were collected during the lean season, while in the present study, data collection was performed during the harvest period. The trend is the same for the other food security indicators. It therefore follows that food security indicators are sensitive to the economic situation.

Compared to the share of food expenditure in total expenditure, the results revealed that more than half of households (58%) spend 50% to more than 75% of their budget on food expenditure. In general, the more food insecure a household is, the greater the share of expenditure devoted to food. Thus, within these households, the greater the proportion of expenditure on food, the greater the risk of food insecurity. A household whose financial resources are mainly devoted to food has a high risk of food insecurity. It also has a very low resilience capacity in the face of a shock, especially if it affects livelihoods. Other essential needs (education, health, leisure, etc.) are marginalized in favor of food. Data from Allen Thomas and Philipp Heinrigs [40] in their study on “New opportunities in the West African food economy” confirm these results. They showed that the share of total household expenditure devoted to food was estimated at 52%. This indicates the significant share of income allocated to food. One approach to solving this situation would be to invest more in people’s access to stable jobs, such as salaried employment, and in facilitating the creation of small- and medium-sized enterprises.

Coping strategies developed by households

To cope with their food access difficulties, households rely on survival strategies to varying degrees. The reduced food strategy index (rCSI) showed that 39% of households resorted to food strategies considered stressful and 9% adopted crisis and severe strategies. These results corroborate those of FAO and WFP [43] as part of their study on monitoring agricultural livelihoods and food and nutritional security in Togo. Their work showed the same trend of a significant proportion of households located in Grand Lome who used crisis strategies the most (62.2%). Furthermore, our analyses showed that nearly half of households develop strategies based on their livelihoods to feed themselves. Thus, 40% of households resorted to stress strategies (using savings, borrowing money, buying food on credit, or borrowing food), 21% of households used crisis strategies (reducing health and education expenses and selling household assets), and 2.9% adopted emergency strategies (begging, migrating the entire household, etc.). This result is confirmed by that of UNICEF, FAO, and WFP [44] in their study on the “impact of rising prices on food security of populations in urban areas in Pikine-Kaolack-Ziguinchor in Senegal”. Their study demonstrated that households have adopted several strategies to cope with the crisis of rising food prices, most of which are presented as follows: obtaining more credit and to some extent financial decapitalization, using savings, reducing expenses in sectors such as health, clothing, and ceremonies, and the sale of jewelry.

### 4.3. Impact on the Nutritional Status of Children Aged 6 to 59 Months

These food consumption and adaptation strategies are not without consequences on the nutritional status of households in Grand Lome. Insufficient or inadequate food intake implies that there are not enough nutrients to build tissues and growth, not enough nutrients to produce antibodies (AC), which can lead to a decrease in immunity. The results indicate that 2.6% of children under 59 months have global acute malnutrition, of which 1.4% are moderately malnourished and 1.2% are severely malnourished, as evidenced by CILSS, FSRP, and DSID [42] in their work on food and nutrition security for the 2022-2023 agricultural campaign. Their results indicate that 2.4% of children aged 6–59 months suffer from global acute malnutrition in the Maritime region of Togo. This proportion of children in a state of global acute malnutrition is below the critical threshold and we can deduce that the food situation at the household level in Grand Lome impacts the nutritional situation of children aged 6–59 months but not significantly.

Study limitations

This study has been prepared and conducted with the utmost rigor. However, it does have some limitations, as follows:

♦ In relation to the data collection period: Although this study has produced some interesting results, it is necessary to bear in mind that some results may be influenced by seasonal factors linked to the data collection period. The data were collected in a post-harvest period (a period of abundance of food products). The situation could be worse during the lean season (June to August) when food prices take a turn for the worse;
♦ With regard to the degree of precision: The results of the data collected from households are precise enough (8.5%) to provide estimates for the prefectures and Greater Lomé. However, the results cannot be estimated at a national level, given the relatively small sample size (310 households);
♦ With regard to data collection: It is true that professional interviewers were recruited for data collection and that they had a good understanding of the French version of the questionnaire, as well as a good command of the local languages spoken in their areas of assignment. In addition, they had been given training, including sessions simulating data collection tools in local languages, in order to reduce any bias inherent in misinterpreting questions or concepts. However, it is possible that some mistakes were made when translating the questionnaire into local languages, as it was in French and had to be administered most of the time in local languages.

## 5. Conclusions

The analysis of food and nutrition security in relation to population and spatial growth is based on food security and nutrition indicators. Despite the availability of food and its accessibility in the markets of Grand Lome, household food security is compromised. The results obtained show that the main method of acquiring food products remains purchasing at markets or in shops. However, despite the good availability of food on urban markets, households in Grand Lome (49%) do not have good dietary diversity and 48% have adopted stress and crisis strategies to access food. Similarly, the prevalence of global acute malnutrition concerns 2.8% of children between 6 and 59 months of age. In addition, households concentrate their food consumption on tubers and cereals, proteins unlike legumes, fruits, and dairy products. It was noted that households headed by women have a better food consumption score compared to households headed by their male counterparts. This situation would be linked to the sensitivity of women to issues relating to food in households. The prefecture with the most households with low dietary diversity is that of Agoè-Nyivé (72.4%). This observation reflects the predominance of precarious neighborhoods and migrants in the prefecture of Agoé-Nyivé.

In view of the results obtained from this study, some suggestions are formulated here in order to make our sustainable contribution to the food and nutritional security of urban households. These following suggestions are addressed to the various stakeholders in food security:

- Contribute to facilitating access to food products by urban households, especially the most vulnerable, by pooling efforts in order to implement joint solutions to the widespread rise in food prices, the high cost of living and the major food and nutritional crisis facing the region and particularly Greater Lomé through access to stable jobs such as salaried employment, facilities in the creation of small- and medium-sized enterprises, and the subsidization of basic food product prices;
- Promote local dishes by proposing culinary recipes that will help improve household consumption and food diversity. This can be achieved by improving the production of low-consumption food groups, strengthening marketing and distribution systems gains, and raising people’s awareness of their nutritional importance;
- Continue the monitoring of food security, livelihoods, and population resilience in urban areas by state technical services in order to facilitate alerts in the event of a food crisis situations.

In terms of prospects, it would be desirable for the present study to be repeated for the study area during the lean season (June, July, and August), in order to see the evolution and severity of the state of food and nutritional indicators over time, and to assess the impact of rapid demographic and spatial growth on household food and nutritional security. These measurements will enable policy-makers and development stakeholders to identify levers for improving food systems in Grand Lome, Togo, and cities across sub-Saharan Africa.

## Figures and Tables

**Table 1:** Sample size by prefecture, by municipality, and by district.

Region	Prefectures	Size of Household to Be Surveyed	Municipalities to Be Investigated	Pop 2022	Average Household Size	Number of Households per Municipality	Weight/ Commune	Number of Households Surveyed	Number of Districts to Be Surveyed (30%)
Grand Lome	Agoé-Nyivé	152	Agoé-Nyivé 1	396,544	4.5	88,121	0.57	87	12
			Agoé-Nyivé 2	159,595	4.5	35,466	0.23	35	6
			Agoé-Nyivé 6	136,450	4.5	30,322	0.20	30	4
			Total	692,589	4.5	153,909	1	152	22
	Golfe	158	Gulf 3	77,825	4.5	17,294	0.11	17	3
			Gulf 4	142,326	4.5	31,628	0.19	30	8
			Gulf 6	196,005	4.5	43,557	0.26	42	2
			Gulf 7	324,260	4.5	72,058	0.44	69	7
			Total	740,416	4.5	164,537	1	158	20
TOTAL								310	

Source: Field work 2023 based on RGPH-5 data, 2022.

**Table 2:** Food consumption score (FCS) thresholds.

Thresholds (SCA)	Household Food Consumption Profile	Meaning of the Different Classes
0–21	Poor	Households that do not consume staple foods and vegetables every day and that never or very rarely consume protein-rich foods such as meat or milk.
21.5–35	Limit	Households that consume staple foods and vegetables every day, accompanied by oil and pulses a few times a week.
Greater than or equal to 35.5	Acceptable	Households that consume staple foods and vegetables daily, frequently accompanied by oil, vegetables, and occasionally meat, fish and dairy products.

Source: CILSS, 2019.

**Table 3:** Household Dietary Diversity Score (HDDS) thresholds.

Thresholds (SDAM)	Household Dietary Diversity Profile
1 to 3 groups	Low diversity
4 to 5 groups	Average diversity
≥6 groups	High diversity

Source: CILSS, 2019.

**Table 4:** Share of food expenditure.

Domain		Indicator	Food Safety (1)	Food Safety Limit (2)	Moderate Food Insecurity (3)	Severe Food Insecurity (4)
Survivability	Income status	Share of food expenditure	<50%	50–<65%	65–<75%	≥75%

Source: CARI, 2014.

**Table 5:** Thresholds of the dietary adaptation strategies index (rCSI).

Weighted Score (rCSI)	Profile of Household Food Adaptation Strategies
0–3	Minimal
4–18	Stress
≥19	Crisis

Source: CILSS, 2019.

**Table 6:** ISAME indicator thresholds.

ScoreISAME	Livelihood Strategies Profile
0	Without strategies
≥1	Stress
≥10	Crisis
≥100	Emergency

Source: CILSS, 2019.

**Table 7:** Classification of MAG/PB.

	Global Acute Malnutrition (GAM)	
Clues	Moderate Acute Malnutrition (MAM)	Severe Acute Malnutrition (SAM)
MUAC	MUAC between 11.5 cm and 12.5 cm	<11.5 cm
Edema		Bilateral

Source: CILSS, 2019.

**Table 8:** Evolution of the income of the households surveyed (compared to the same period of the last five years).

Evolution of Household Income	Decrease (−54%)	No Change (27%)	Increase (19%)
Households affected by main activity	- Trade (70.0%) - Tailor, dressmaker, hairdresser… (54.6%) - Retired (57.1%) - Lawyer, surveyor, bailiff (66.7%) - Transport including motorcycle taxi (64.5%)	- Civil servant (62.5%) - Private administration agent (46.9%)	
Households affected according to the Prefecture	Agoè-Nyivé (50%) Gulf (55%)	-	
Households affected by gender of CM	Male (48.79%) Female (67.74%)		

Source: Field survey, November 2023.

**Table 9:** Household food consumption score.

Levels		SCA						
		Acceptable		Limit		Poor		Signification (*p*)
		Headcount	(%)	Headcount	(%)	Headcount	(%)	
Prefectures	Agoè-Nyivé	128	84.2%	23	15.1%	1	0.7%	0.000
	Golfe	101	63.9%	48	30.4%	9	5.7%	
Gender of head of household Grand Lome	Male	183	73.8%	58	23.4%	7	2.8%	0.686
	Female	46	74.2%	13	21.0%	3	4.8%	
	Total	229	73.9%	71	22.9%	10	3.2%	

Source: Field survey, November 2023.

**Table 10:** Household Dietary Diversity Score (HDDS).

Levels		SDAM						
		High Diversity		Average Diversity		Low Diversity		Signification (*p*)
		Headcount	(%)	Headcount	(%)	Headcount	(%)	
Prefectures	Agoè-Nyivé	42	27.6%	91	59.9%	19	12.5%	0.955
	Golfe	44	27.8%	96	60.8%	18	11.4%	
Gender of head of household Grand Lome	Male	72	29.0%	145	58.5%	31	12.5%	
	Female	14	22.6%	42	67.7%	6	9.7%	0.410
	Total	86	27.7%	187	60.3%	37	11.9%	

Source: Field survey, November 2023.

**Table 11:** Reduced Food Strategies Index (rCSI).

Levels		rCSI						
		MINIMAL		STRESS		CRISIS		Signification (*p*)
		Headcount	(%)	Headcount	(%)	Headcount	(%)	
Prefectures	Agoè-Nyivé	88	57.9%	55	36.2%	9	5.9%	0.054
	Golfe	73	46.2%	66	41.8%	19	12.0%	
Gender of head of household Grand Lome	Male	133	53.6%	95	38.3%	20	8.1%	0.343
	Female	28	45.2%	26	41.9%	8	12.9%	
	Total	161	51.9%	121	39.0%	28	9.0%	

Source: Field survey, November 2023.

**Table 12:** Livelihood-based coping strategies.

Levels		ISAME								Signification (*p*)
		Without Strategy		Stress		Crisis		Emergency		
		Headcount	(%)	Headcount	(%)	Headcount	(%)	Headcount	(%)	
Prefectures	Agoè-Nyivé	52	34.2%	63	41.4%	36	23.7%	1	0.7%	0.083
	Golfe	60	38.0%	61	38.6%	29	18.4%	8	5.1%	
Gender of head of household Grand Lome	Male	90	36.3%	103	41.5%	49	19.8%	6	2.4%	0.457
	Female	22	35.5%	21	33.9%	16	25.8%	3	4.8%	
	Total	112	36.1%	124	40.0%	65	21.0%	9	2.9%	

Source: Field survey, November 2023.

**Table 13:** Prevalence of acute malnutrition PB by prefecture and in Grand Lome.

Levels	Headcount	Global	Moderate	Severe
		Prevalence of Global Acute Malnutrition MUAC < 125 mm (95% CI)	MUAC < 125 and MUAC >= 115 mm (95% CI)	Prevalence of Severe Malnutrition MUAC < 115 mm (95% CI)
Golfe	Total: 152	1.3% (0.2–6.1)	1.3% (0.4–4.7)	0% (0.0–2.5)
Agoe-Nyive	Total: 151	2.10% (0.7–5.7)	1.3% (0.4–4.7)	0.7% (0.1–3.7)
Grand Lome	Boys: 137	1.6 (1.0–2.5)	1.0 (0.6–1.8)	0.6 (0.3–1.1)
	Girls: 166	2.4 (1.5–3.5)	1.8 (1.2–2.8)	0.6 (0.3–1.3)
	Total: 303	2.6 (1.5–2.8)	1.4 (0.3–2.0)	1.2 (1.1–4.4)

Source: Field survey, November 2023.

## Data Availability

The original contributions presented in the study are included in the article, further inquiries can be directed to the corresponding author.

## Appendix A

**Table A1:** Score de consommation alimentaire des ménages (CSA).

Caractéristiques Socioéconomiques		Pauvre	Limite	Acceptable	Total	Signification Asymptotique (Bilatérale		
PREFECTURE	GOLFE	5.7%	30.4%	63.9%	100.0%	18.277	0.000	***
	AGOE-NYIVE	0.7%	15.1%	84.2%	100.0%			
8.2 Sexe du chef de ménage	Masculin	2.8%	23.4%	73.8%	100.0%	0.753	0.686	
	Féminin	4.8%	21.0%	74.2%	100.0%			
Tranche d’âge	<=25	0.0%	40.0%	60.0%	100.0%	10.774	0.375	
	[26–35]	2.2%	21.3%	76.4%	100.0%			
	[36–45]	2.7%	16.8%	80.5%	100.0%			
	[46–55]	6.0%	29.9%	64.2%	100.0%			
	[56–65]	5.9%	23.5%	70.6%	100.0%			
	66+	0.0%	35.7%	64.3%	100.0%			
Statut matrimonial du chef de ménage	Marié(e)	3.0%	21.6%	75.4%	100.0%	3.393	0.758	
	Séparé(e)/Divorcé(e)	3.8%	23.1%	73.1%	100.0%			
	Veuf(ve)	3.2%	22.6%	74.2%	100.0%			
	Célibataire	4.8%	38.1%	57.1%	100.0%			
Nombre totale de personnes dans le ménage	1 à 3 personnes	5.0%	26.3%	68.8%	100.0%	4.002	0.676	
	4 à 6 personnes	2.2%	21.1%	76.8%	100.0%			
	7 à 9 personnes	5.3%	26.3%	68.4%	100.0%			
	10 personnes et plus	0.0%	14.3%	85.7%	100.0%			
2.1-Quels sont les chocs les plus graves auxquels le ménage a été confronté au cours des 12 derniers mois?	Aucun_choc	2.4%	21.2%	76.3%	100.0%	22.306	0.034	**
	Maladie_deces	5.0%	30.0%	65.0%	100.0%			
	Perte_emploi	50.0%	50.0%	0.0%	100.0%			
	Prix_eleve_aliment	0.0%	44.4%	55.6%	100.0%			
	Prix_eleve_carburant	6.9%	24.1%	69.0%	100.0%			
	Inondation_pluies_diluviennes	0.0%	33.3%	66.7%	100.0%			
	Incendie	0.0%	0.0%	100.0%	100.0%			
3.1-Quelle est la principale activité génératrice de revenu?	Commerce	2.5%	21.3%	76.3%	100.0%	16.055	0.310	
	Artisanat (Tailleur, couturier, coifeuse…)	6.2%	22.7%	71.1%	100.0%			
	Agent de la fonction publique	4.2%	4.2%	91.7%	100.0%			
	Agent d’une administration privée	3.1%	18.8%	78.1%	100.0%			
	Retraité	0.0%	21.4%	78.6%	100.0%			
	Profession libérale	0.0%	33.3%	66.7%	100.0%			
	Transport (y compris taxi moto)	0.0%	38.5%	61.5%	100.0%			
	Autre activité	0.0%	28.6%	71.4%	100.0%			
3.3-Avez-vous une activité secondaire génératrice de revenu?	Oui	5.7%	19.3%	75.0%	100.0%	3.009	0.222	
	Non	2.3%	24.3%	73.4%	100.0%			
Total		3.2%	22.9%	73.9%	100.0%			

** Significatif à 5%; *** Significatif à 1%.

**Table A2:** Score de Diversité alimentaire des ménages (SDAM).

Caractéristiques Socioéconomiques		Groupe de Score de Diversité Alimentaire			Total	Tests du Khi-Carré		
		Diversité Faible	Diversité Moyenne	Diversité Élevée		Valeur	ddl	Signification Asymptotique (Bilatérale)
Préfecture	GOLFE	11.4%	60.8%	27.8%	100.0%	0.091	2	0.955
	AGOE-NYIVE	12.5%	59.9%	27.6%	100.0%			
Sexe du chef de ménage	Masculin	12.5%	58.5%	29.0%	100.0%	1.782	2	0.410
	Féminin	9.7%	67.7%	22.6%	100.0%			
Tranche d’âge	<=25		90.0%	10.0%	100.0%	12.803	10	0.235
	[26–35]	10.1%	66.3%	23.6%	100.0%			
	[36–45]	13.3%	51.3%	35.4%	100.0%			
	[46–55]	16.4%	59.7%	23.9%	100.0%			
	[56–65]	11.8%	64.7%	23.5%	100.0%			
	66+		71.4%	28.6%	100.0%			
Statut matrimonial du chef de ménage	Marié(e)	12.1%	59.1%	28.9%	100.0%	1.542	6	0.957
	Séparé(e)/Divorcé(e)	15.4%	57.7%	26.9%	100.0%			
	Veuf(ve)	9.7%	67.7%	22.6%	100.0%			
	Célibataire	9.5%	66.7%	23.8%	100.0%			
Niveau d’instruction du chef de ménage **	Aucun	16.0%	80.0%	4.0%	100.0%	20.693	10	0.023
	Alphabétisé		100.0%		100.0%			
	Coranique/Islamique		50.0%	50.0%	100.0%			
	Primaire	14.7%	67.6%	17.6%	100.0%			
	Secondaire	12.5%	57.9%	29.6%	100.0%			
	Supérieur/Universitaire	6.9%	50.0%	43.1%	100.0%			
Nombre totale de personnes dans le ménage	1 à 3 personnes	17.5%	61.3%	21.3%	100.0%	10.369	6	0.110
	4 à 6 personnes	8.1%	61.6%	30.3%	100.0%			
	7 à 9 personnes	21.1%	50.0%	28.9%	100.0%			
	10 personnes et plus		71.4%	28.6%	100.0%			
Quels sont les chocs les plus graves auxquels le ménage a été confronté au cours des 12 derniers mois?	Aucun_choc	12.2%	60.0%	27.8%	100.0%	12.361	12	0.417
	Maladie_deces	15.0%	70.0%	15.0%	100.0%			
	Perte_emploi	50.0%	50.0%		100.0%			
	Prix_eleve_aliment		88.9%	11.1%	100.0%			
	Prix_eleve_carburant	10.3%	51.7%	37.9%	100.0%			
	Inondation_pluies_diluviennes		33.3%	66.7%	100.0%			
	Incendie		50.0%	50.0%	100.0%			
Quelle est la principale activité génératrice de revenu?	Commerce	8.8%	63.8%	27.5%	100.0%	16.597	14	0.278
	Artisanat (Tailleur, couturier, coiffeuse…)	16.5%	56.7%	26.8%	100.0%			
	Agent de la fonction publique	4.2%	50.0%	45.8%	100.0%			
	Agent d’une administration privée	15.6%	50.0%	34.4%	100.0%			
	Retraité		78.6%	21.4%	100.0%			
	Profession libérale (Avocat, géomètre, Huissier…)		33.3%	66.7%	100.0%			
	Transport (y compris taxi moto)	12.8%	66.7%	20.5%	100.0%			
	Autre activité	14.3%	71.4%	14.3%	100.0%			
Avez-vous une activité secondaire génératrice de revenu ? *	Oui	18.2%	58.0%	23.9%	100.0%	4.797	2	0.091
	Non	9.5%	61.3%	29.3%	100.0%			

* Significatif à 10%; ** Significatif à 5%.

**Table A3:** Indice réduit de Stratégies alimentaires (rCSI).

Caractéristiques Socioéconomiques		Groupe rCSI			Total	Tests du Khi-Carré		
		Minimal	Stress	Crise		Valeur	ddl	Signification Asymptotique (Bilatérale)
Préfecture *	GOLFE	46.2%	41.8%	12.0%	100.0%	5.855	2	0.054
	AGOE-NYIVE	57.9%	36.2%	5.9%	100.0%			
8.2-Sexe du chef de ménage	Masculin	53.6%	38.3%	8.1%	100.0%	2.138	2	0.343
	Féminin	45.2%	41.9%	12.9%	100.0%			
Tranche d’âge *	<=25	50.0%	40.0%	10.0%	100.0%	17.164	10	0.071
	[26–35]	60.7%	34.8%	4.5%	100.0%			
	[36–45]	53.1%	39.8%	7.1%	100.0%			
	[46–55]	37.3%	43.3%	19.4%	100.0%			
	[56–65]	64.7%	29.4%	5.9%	100.0%			
	66+	42.9%	50.0%	7.1%	100.0%			
Statut matrimonial du chef de ménage	Marié(e)	52.2%	39.7%	8.2%	100.0%	4.255	6	0.642
	Séparé(e)/Divorcé(e)	46.2%	38.5%	15.4%	100.0%			
	Veuf(ve)	45.2%	45.2%	9.7%	100.0%			
	Célibataire	66.7%	23.8%	9.5%	100.0%			
Niveau d’instruction du chef de ménage **	Aucun	28.0%	48.0%	24.0%	100.0%	22.071	10	0.015
	Alphabétisé		100.0%		100.0%			
	Coranique/Islamique	83.3%	16.7%		100.0%			
	Primaire	42.6%	44.1%	13.2%	100.0%			
	Secondaire	53.9%	38.8%	7.2%	100.0%			
	Supérieur/Universitaire	65.5%	31.0%	3.4%	100.0%			
Nombre totale de personnes dans le ménage **	1 à 3 personnes	60.0%	37.5%	2.5%	100.0%	20.187	6	0.003
	4 à 6 personnes	53.0%	38.9%	8.1%	100.0%			
	7 à 9 personnes	34.2%	42.1%	23.7%	100.0%			
	10 personnes et plus	28.6%	42.9%	28.6%	100.0%			
Quels sont les chocs les plus graves auxquels le ménage a été confronté au cours des 12 derniers mois?	Aucun_choc	52.7%	38.0%	9.4%	100.0%	18.429	12	0.103
	Maladie_deces	40.0%	60.0%		100.0%			
	Perte_emploi	50.0%		50.0%	100.0%			
	Prix_eleve_aliment	55.6%	44.4%		100.0%			
	Prix_eleve_carburant	55.2%	37.9%	6.9%	100.0%			
	Inondation_pluies_diluviennes	66.7%		33.3%	100.0%			
	Incendie		50.0%	50.0%	100.0%			
Quelle est la principale activité génératrice de revenu? **	Commerce	53.8%	40.0%	6.3%	100.0%	31.111	14	0.005
	Artisanat (Tailleur, couturier, coiffeuse…)	44.3%	44.3%	11.3%	100.0%			
	Agent de la fonction publique	87.5%	12.5%		100.0%			
	Agent d’une administration privée	59.4%	28.1%	12.5%	100.0%			
	Retraité	50.0%	42.9%	7.1%	100.0%			
	Profession libérale (Avocat, géomètre, Huissier…)	66.7%	33.3%		100.0%			
	Transport (y compris taxi moto)	46.2%	51.3%	2.6%	100.0%			
	Autre activité	38.1%	33.3%	28.6%	100.0%			
Avez-vous une activité secondaire génératrice de revenu?	Oui	47.7%	38.6%	13.6%	100.0%	3.308	2	0.191
	Non	53.6%	39.2%	7.2%	100.0%			
Total		51,9%	39.0%	9.0%	100.0%			

* Significatif à 10%; ** Significatif à 5%.

**Table A4:** Stratégies d’adaptation basées sur les moyens d’existence.

Caractéristiques Socioéconomiques		ISAME					Tests du Khi-Carré		
		Sans Strategie	Stress	Crise	Urgence		Valeur	ddl	Signification Asymptotique (Bilatérale)
Préfecture *	GOLFE	38.0%	38.6%	18.4%	5.1%	100.0%	6.688	3	0.083
	AGOE-NYIVE	34.2%	41.4%	23.7%	0.7%	100.0%			
Total		36,1%	40.0%	21.0%	2.9%	100.0%			
Sexe du chef de ménage	Masculin	36.3%	41.5%	19.8%	2.4%	100.0%	2.602	3	0.457
	Féminin	35.5%	33.9%	25.8%	4.8%	100.0%			
Tranche d’âge **	<=25	60.0%	20.0%	10.0%	10.0%	100.0%	28.588	15	0.018
	[26–35]	30.3%	50.6%	18.0%	1.1%	100.0%			
	[36–45]	38.9%	37.2%	17.7%	6.2%	100.0%			
	[46–55]	29.9%	34.3%	35.8%		100.0%			
	[56–65]	52.9%	35.3%	11.8%		100.0%			
	66+	42.9%	42.9%	14.3%		100.0%			
Statut matrimonial du chef de ménage	Marié(e)	34.9%	42.2%	20.3%	2.6%	100.0%	14.645	9	0.101
	Séparé(e)/Divorcé(e)	30.8%	50.0%	11.5%	7.7%	100.0%			
	Veuf(ve)	35.5%	29.0%	35.5%		100.0%			
	Célibataire	57.1%	19.0%	19.0%	4.8%	100.0%			
Niveau d’instruction du chef de ménage	Aucun	36.0%	36.0%	20.0%	8.0%	100.0%	19.033	15	0.212
	Alphabétisé			100.0%		100.0%			
	Coranique/Islamique	50.0%	50.0%			100.0%			
	Primaire	25.0%	39.7%	32.4%	2.9%	100.0%			
	Secondaire	38.2%	43.4%	15.8%	2.6%	100.0%			
	Supérieur/Universitaire	43.1%	32.8%	22.4%	1.7%	100.0%			
Nombre totale de personnes dans le ménage	1 à 3 personnes	43.8%	38.8%	15.0%	2.5%	100.0%	13.485	9	0.142
	4 à 6 personnes	36.2%	38.9%	22.2%	2.7%	100.0%			
	7 à 9 personnes	18.4%	47.4%	31.6%	2.6%	100.0%			
	10 personnes et plus	42.9%	42.9%		14.3%	100.0%			
Quels sont les chocs les plus graves auxquels le ménage a été confronté au cours des 12 derniers mois? **	Aucun_choc	32.2%	42.4%	22.4%	2.9%	100.0%	32.285	18	0.020
	Maladie_deces	40.0%	50.0%	10.0%		100.0%			
	Perte_emploi	50.0%			50.0%	100.0%			
	Prix_eleve_aliment	44.4%	22.2%	33.3%		100.0%			
	Prix_eleve_carburant	58.6%	24.1%	13.8%	3.4%	100.0%			
	Inondation_pluies_ diluviennes	66.7%	33.3%			100.0%			
	Incendie	50.0%		50.0%		100.0%			
Quelle est la principale activité génératrice de revenu? **	Commerce	46.3%	27.5%	21.3%	5.0%	100.0%	35.654	21	0.024
	Artisanat (Tailleur, couturier, coifeuse…)	26.8%	45.4%	23.7%	4.1%	100.0%			
	Agent de la fonction publique	70.8%	25.0%	4.2%		100.0%			
	Agent d’une administration privée	34.4%	43.8%	21.9%		100.0%			
	Retraité	28.6%	50.0%	21.4%		100.0%			
	Profession libérale (Avocat, géomètre, Huissier…)	66.7%	33.3%			100.0%			
	Transport (y compris taxi moto)	17.9%	53.8%	28.2%		100.0%			
	Autre activité	38.1%	42.9%	14.3%	4.8%	100.0%			
Avez-vous une activité secondaire génératrice de revenu?	Oui	36.4%	42.0%	19.3%	2.3%	100.0%	0.458	3	0.928
	Non	36.0%	39.2%	21.6%	3.2%	100.0%			

* Significatif à 10%; ** Significatif à 5%.
